# Optimization of Cultural Conditions for* Bacillus megaterium* Cultured in* Agaricus bisporus* Industrial Wastewater

**DOI:** 10.1155/2018/8106245

**Published:** 2018-12-26

**Authors:** Jiafu Huang, Danfeng Zhang, Yixin Ou, Guoguang Zhang, Linhua Zheng, Lizhuan Lin, Xiaomei Ye, Xiaofeng Zhu, Yutian Pan

**Affiliations:** ^1^Engineering Technological Center of Mushroom Industry, Minnan Normal University, Zhangzhou, Fujian 363000, China; ^2^School of Life Sciences & Biotechnology College, Minnan Normal University, Zhangzhou, Fujian 363000, China; ^3^State Key Laboratory of Microbial Metabolism and School of Life Sciences & Biotechnology, Shanghai Jiao Tong University, Shanghai, 200240, China

## Abstract

The aim of this study was to optimize the cultural conditions for* Bacillus megaterium* using* Agaricus bisporus* industrial wastewater as nature culture through response surface methodology. In our present study, we analyzed the total number of living* B. megaterium* in the fermentation broth using multispectral imaging flow cytometry. Plackett-Burman design was applied to evaluate the effects of six variables, namely, initial pH, industrial wastewater solubility, rotating speed, culture temperature, inoculum size, and loading volume. Loading volume, initial pH, and culture temperature were found to influence the biomass of* B. megaterium* significantly and were further optimized by Box-Behnken design. After verification test, the optimum fermentation conditions of* B. megaterium* using the* A. bisporus* processing wastewater as nature culture media were obtained as follows: initial pH of 7.4, culture temperature of 25°C, loading volume of 40 mL/250 mL, culture time of 24 h, industrial wastewater solubility of 1%, rotating speed of 200 rpm, and inoculum size of 8%. The predicted optimum model's value was 8.88 × 10^8^ Obj/mL and the average experimental value was 9.03 ± 0.02 × 10^8^ Obj/mL, which met the national microbial fertilizers' standard. Furthermore, the field experiment results showed that the fermentation broth of* B. megaterium *could significantly improve the yield of* Spinacia oleracea L.*

## 1. Introduction

Along with the extensive use of fertilizers and pesticides in agricultural production, leading to serious ecological environment pollution and sharp fall of crop quality, microbial fertilizers more and more get people's attention.* B. megaterium* is a kind of plant growth promotion rhizobacteria and one common bacterium of microbial fertilizer, can degrade lecithin, organic phosphorus, and inorganic phosphorus in the soil to plants, can directly adsorb [1], can stimulate plant growth and alleviate phytotoxicity of pollutants [2–4], and also can improve soil fertility and crop disease resistance [5, 6].

*A. bisporus* is commonly known as the white button mushroom and is commonly referred to as a “mushroom”. It is one of the most economically important and widely produced mushrooms in the world. Mushrooms are primarily stored in cans and traded across the world. During the processes of the mushroom cans production, washing, preboiling, and canning, fresh mushrooms produce a large quantity of wastewater. The mushroom industrial wastewater has high BOD and COD content, of which the content of COD is 540.29 g/L, which is 13.07 times higher than the national three level emission standard [7]. The discharge of the wastewater into the surrounding areas is a cause of environmental pollution, so the wastewater discharged from mushroom processing industries has become a large burden to the environment. Developing methods for utilizing and disposing the mushroom processing wastewater is an important priority.

The wastewater from these processes contains a large amount of soluble components of the mushroom [7, 8]. Crude polysaccharides and 4 novel components (Abnp1001, Abnp1002, Abap1001, and Abap1002) were successfully obtained from the mushroom wastewater. The extraction of polysaccharides from the industrial mushroom wastewater yielded 3.30 g/g, 0.989 mg/g, 1.849 mg/g, 0.128 mg/g, and 0.68 mg/g, respectively. [7] There are 32.16% content of D-mannitol in the concentrated* A. bisporus *industrial wastewater [8]. And moreover, the test report of product of* A. bisporus* industrial wastewater in the processing enterprises (Fujian KEREN Biological Co., Ltd.) showed that there was 18.9% proteins in their product, which could provide sufficient carbon and nitrogen source for microbiological [9] and plants growth [10]. Now therefore, we attempt to search an optimal fermentation condition of* B. megaterium* fermented via shaking culture by using mushroom processing wastewater as a natural medium in our present study, which provided theoretical support for the development of the downstream industry of* A. bisporus*.

## 2. Materials and Methods

### 2.1. Strains and Growth Conditions

*B. megaterium* (GIM 1.270) was purchased from the Guangdong Culture Collection Center.

Seed medium is as follows: peptone 5 g, beef extract 3 g, NaCl 5 g, distilled water 1 L, pH 6.2, boiled and thawed, aliquoted and sterilized at 121°C for 15 min.

Preparation of seed suspension is as follows:* B. megaterium* which has been activated with vaccination loop was picked, with access to sterilized seed liquid medium containing 100 mL flask made of bacterial suspension, and placed in a shaker (30°C, 150 rpm) for 24 h.

*Plackett-Burman design. *Plackett-Burman design methods can be used in the screening of the key influential factors compared with other statistical designs on the experimental response [11]. Six variables (industrial wastewater solubility, initial pH, inoculum dose, loaded liquid, culture temperature, and shaking speed) were selected in the screening approach with Plackett-Burman experimental design, suggesting 12 trial runs, and the total number of viable bacteria was selected as the observed response to determine the effects of the variables studied [12]. To increase the response, each factor selected was tested in two levels, high levels (+1) and low levels (-1). The Plackett-Burman experiments design was formulated for six factors that affect the biomass of* B. megaterium* using the Design-Expert version 8.0.6 software (Stat-Ease, Inc., Minneapolis, MN, USA). The factors and levels of Plackett-Burman design shown in Table 1 were determined based on the one-factor-at-a-time experiments.

*Steepest ascent design. *The steepest ascent experiment was performed to move the experimental region of the response in the direction of the optimum after having identified the three most significant variables through the Plackett-Burman design [13]. The path was initiated from the design center of the factorial design (the screening design) and receded when no further improvement in the response could be achieved. When the maximum value was gained, the point could be considered as the center point for the optimization experimental design [14].  Table 2 summarizes the steepest ascent experimental design, the variables, and their values.

*Box-Behnken design. *The response surface methodology is a very practical and valuable tool for fermentation industry to improve product yield and minimize by‐products as well as reduce overall manufacturing costs [15], and it is possible for response surface methodology to derive an expression for performance measurement on the basis of the response values obtained from experiments at a particular combination of input variables [16]. In the present study, by employing Box-Behnken design and response surface methodology, the effects of the three independent variables (loaded liquid, 15-60 mL/250mL; liquid pH, 7.0-8.0; culture temperature, 24-32°C) and three levels (high, middle, and low) on the response (the total number of living bacteria) were investigated to determine the optimal conditions, which maximized the biomass of* B. megaterium* from shake cultivation. The Box-Behnken design comprised 17 experiments with five center points (to allow for estimation of pure error) and facilitated calculations of response function at intermediate levels, fitting a second-order response surface.  Table 3 showed the variables and their values and the experimental design.

*Field experiment. *The well-developed and proportioned seeds of* Spinacia oleracea L. *germinating on the moist and 20°C condition were selected to sow at 2 cm depth. After sprouting 5 days, the plants of* S. oleracea L.* were transplanted to 7 seedling plates with the same plain soil and vertically placed into the hole, two plans per hole. The 7 seeding plates of* S. oleracea L.* were treated with different dose of fermentation broth of* B. megaterium* cultured on the optimized condition. Seeding plate 1 was the control group and only treated with the same dose of water in the vegetation process. Seeding plate 2 was applied with one time of the fermentation broth after transplanting plant survived. Seeding plate 3 was, respectively, treated one time at the surviving time and during middle time of upgrowth. Seeding plate 4 was treated 3 times: the first two times were the same as seeding plate 3, and the third time came into operation at 10 days before harvest. Seeding plates 5-7 were the industrial wastewater control groups and treated with the same dose of* A. bisporus* industrial wastewater. Seeding plate 5 was applied one time after transplanting plant survived. Seeding plate 6 was, respectively, treated one time at the surviving time and during middle time of upgrowth. Seeding plate 7 was treated 3 times: the first two times were the same as seeding plate 6, and the third time came into operation at 10 days before harvest. The 500 mL water,* A. bisporus *industrial wastewater, and fermentation broth were treated with the 7 seeding plates every time, respectively. During the cultivation, watering was necessary to keep the moist of soil. After transplanting for 30 days,* S. oleracea L.* in the 7 seeding plates was gathered, and its plant height, yield, and leaf area were analyzed. The experiment was repeated in triplicate.

*Determination of the total number of living B. megaterium. *Take the fermentation broth diluted 10 times with PBS, take 1mL diluted solution plus 3*μ*L LIVE/DEAD Baclight™ staining reagent, after mixing, avoid light for 30min, and analyze by multispectral imaging flow cytometry [17]. The PBS solution was used as a flow sheath; 480nm laser was used to collect fluorescence signals and images of 20000 bright field (BF), channel 2 green fluorescence (SYTO 9), and channel 5 red fluorescence (propidium iodide, PI); the SYTO 9 signal is the X axis, the PI signal is the Y axis, and the scatter plot is made to distinguish the total number of the dead and the statistical living bacteria.

## 3. Results and Discussion

### 3.1. The Part Results of the Total Number of Living B. megaterium in the Fermentation Liquid Analyzed by Multispectral Imaging Flow Cytometry

**Figure 1:**
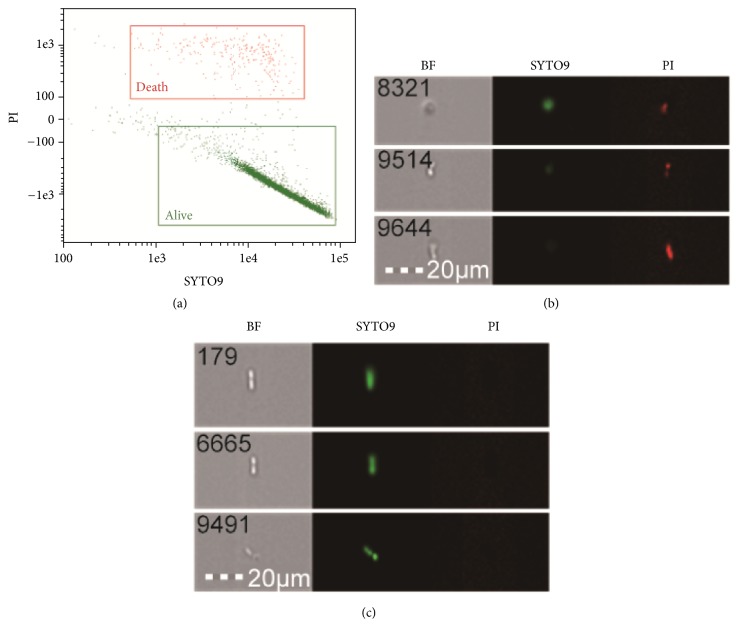
Live and dead* B. megaterium* based on multispectral imaging flow. Note. (a) From the collected images, live and dead* B. megaterium* were visually identified (as indicated by colored crosses) and the tagged populations were gated on the original plot. (b) Images of dead* B. megaterium* in the fermented liquid. (c) Images of live* B. megaterium* in the fermented liquid. Images from within each region were chosen at random. From left to right, bright field, SYTO 9, and PI channel images are displayed.

As shown in Figure 1, two segregated regions were identified and gated (Figure 1(a)): SYTO 9 green-fluorescent nucleic acid stain mixed with the red-fluorescent nucleic acid stain, PI; those stains differ both in their spectral characteristics and in their ability to penetrate healthy bacterial cells [18, 19]. Thus, with an appropriate mixture of the SYTO 9 and PI, bacteria with intact cell membranes stain fluorescent green (Figure 1(c)), whereas bacteria with damaged membranes stain fluorescent red (Figure 1(b)). Clearly we could score which bacteria are dead and which bacteria are alive in this assay.

### 3.2. Plackett-Burman Experiment

Plackett-Burman experimental design matrix and results are shown in Table 4 and analysis of variance is depicted in Table 5. The model F-value of 7.17 in Table 5 implies that the Plackett-Burman experimental model is significant and that there is only a 2.36% chance that a “model F-value” this large could occur due to noise. The* p* values of each factor in Table 5 were used to determine the significance of the screened variables; therefore, Table 5 indicated that three out of six variables,* viz.*, liquid quantity, initial pH, and cultured temperature, had a significant influence on the total number of living bacteria. The Coefficient Estimate of variables displayed the positive or negative effect on the response value. Therefore, loaded liquid showed the highest level of significance with Coefficient Estimate of -1.07 with an F-value of 17.78 and a very low* p *=0.0084 < 0.01, which indicated loaded liquid had the significant positive effect on the biomass of* B. megaterium*. By that analogy, the initial pH exhibited significant positive effects and culture temperature exhibited significant negative effects.

### 3.3. Steepest Ascent Design

According to analysis of variance in Plackett-Burman design, six sets of experiments of the steepest ascent and corresponding experimental results were shown in Table 2. The biomass of* B. megaterium* peaked at the fifth step and no further improvement could be achieved in the response when the loaded liquid was 30 mL/250mL, the initial pH was 7.5, and the culture temperature was 28°C, which suggested that it was proximal to the region of maximum response. Accordingly, these levels of the three factors in the fifth set were considered the center point of Box-Behnken design.

### 3.4. Optimization of Fermentation Parameters by Response Surface Methodology

According to the result of steepest ascent design, the response surface experiments were designed to obtain a second-order polynomial equation consisting of 12 trials plus 5 central points; thus the design matrix of the variables was shown in Table 3 along with the experimental values of response. Through multiple regression analysis of the experimental data, shown in Table 6, the following second-order polynomial equation was derived for the total number of living* B. megaterium* by only considering the significant terms: Y=8.48-1.41*X*_1_+0.24*X*_2_+1.54*X*_3_-0.73*X*_1_*X*_2_+0.092*X*_1_*X*_3_-0.097*X*_2_*X*_3_-2.58*X*_1_^2^-2.59*X*_2_^2^-2.97*X*_3_^2^, where Y is the predicted response of the total number of living* B. megaterium* and* X*_1_,* X*_2_, and* X*_3_ are the coded values of loaded liquid, initial pH, and culture temperature, respectively.

A large model F-value indicates that most of the variation in the response can be explained by a given regression equation. As shown in Table 6, the F-value of model was 86.36, which indicated that the terms in the model had a significant effect on the response, and the* p* < 0.0001 of regression model indicated that the linear relationship between dependent variable and all independent variables was very significant; that is, the experimental method was reliable. The lack of fit for the model was insignificant (*p* < 0.05) with* p* value of 0.1964, suggesting that the experimental data obtained was a good fit with the model. The value of adj-R^2^ (0.9796) suggested that the total variation of 97.96% for the biomass of* B. megaterium* was attributed to the independent variables. The determination coefficient (R^2^=0.9911), which is commonly used to assess the goodness of the model, exhibited an excellent correlation between the experimental and predicted response values. A low CV (CV = 9.20%) value clearly revealed that the deviations between experimental and predicted values were low and it displayed not only a high degree of precision but also high reliability in conducted experiments. Adequate precision measures the signal-to-noise ratio, and a ratio greater than 4 is desirable. In this study, a ratio of 26.229 indicated an adequate signal. Therefore, the quadratic model was selected in this optimization study.

**Figure 2:**
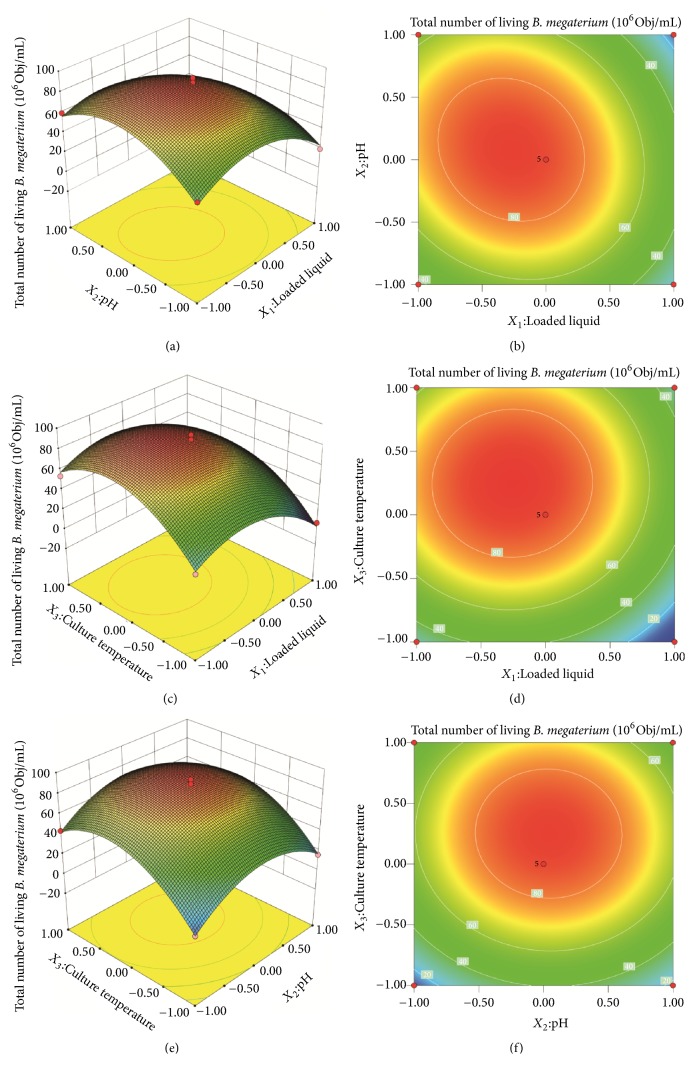
The effect of cross-interaction among load liquid, pH, and culture temperature on total number of alive* B. megaterium*. Note. (a) Response surface plot of effects of interaction between load liquid and pH on total number of alive* B. megaterium*. (b) Contour line of effects of interaction between load liquid and pH on total number of alive* B. megaterium*. (c) Response surface plot of effects of interaction between load liquid and culture temperature on total number of alive* B. megaterium*. (d) Contour line of effects of interaction between load and culture temperature on total number of alive* B. megaterium*. (e) Response surface plot of effects of interaction between pH and culture temperature on total number of alive* B. megaterium*. (f) Contour line of effects of interaction between pH and culture temperature on total number of alive* B. megaterium.*

The optimal levels of each variable for a maximum biomass of* B. megaterium* were determined by creating three-dimensional (3D) response surface plots. Interpretation of the response surface 3D model and contour plot were the graphical representations of regression equation. They provided visual interpretations of the relationship between responses and experimental levels of each variable, and the type of interactions between two test variables [20], as shown in Figure 2, the profiles of the response surfaces between loaded liquid (*X*_1_) and pH (*X*_2_) (Figure 2(a)), loaded liquid (*X*_1_) and temperature (*X*_3_) (Figure 2(c)), pH (*X*_2_) and temperature (*X*_3_) (Figure 2(e)) were all convex with an open downward direction, therefore, indicating a high total number of viable* B. megaterium*. When the loaded liquid (test level is 0) was 30 mL/250mL, the pH was 7.5 (test level is 0), and the culture temperature was 28°C (test level is 0); the total number of living bacteria reached the actual maximum (8.75 ± 0.04, 8.67 ± 0.03, 8.57 ± 0.03 × 10^8^ Obj/mL) closing to the design points, respectively. Furthermore, the shape of the contour plot could explain the significant interaction between two test variables. The contour line (Figure 2(b)) is oval and the interaction between load liquid and pH was significant. However, the contour line (Figures 2(d) and 2(f)) is round and the interactions between loaded liquid and temperature, pH and temperature were not significant.

These observations were also verified through canonical analysis of the response surface. By solving the inverse matrix from the second-order polynomial equation, the optimum values of the test variables were loaded liquid 39.33 mL/250mL, pH 7.36, and temperature 25.01°C, and the predictive value of the total number of viable* B. megaterium* was 8.88 × 10^8^ Obj/mL. To confirm the validity of the model for predicting the maximum total number of viable* B. megaterium*, an additional experiment using this optimum operation conditions was performed under shake-flask culture. The total number of viable* B. megaterium* was 9.05 ± 0.03 × 10^8^ Obj/mL (N = 3), which was 1.02 times the predicted value, suggesting that the model was adequate for reflecting the expected optimization, and the response surface methodology model was satisfactory and accurate.

### 3.5. Field Experiment

**Figure 3:**
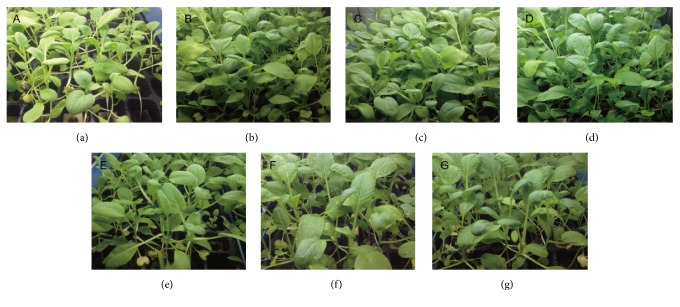
The grown status of spinach. Note. (a) Control group treated with water. (b) Spinach treated with fermentation broth one time. (c) Spinach treated with fermentation broth two times. (d) Spinach treated with fermentation broth three times. (e) Spinach treated with* A. bisporus* industrial wastewater one time. (f) Spinach treated with* A. bisporus* industrial wastewater two times. (g) Spinach treated with* A. bisporus* industrial wastewater three times.

As shown in Table 7, the plant height, yield, and leaf area of* S. oleracea L.* were significantly improved as the times that* S. oleracea L. *was treaded with the fermentation liquid of* B. megaterium* using* A. bisporus* industrial wastewater as culture increased. Although the yield of* S. oleracea* in the* A. bisporus* industrial wastewater control group was significant compared with the control group, plant height, yield, and leaf area in groups treated with fermentation broth of* B. megaterium* were more conspicuously increasing. As shown in Figure 3, the grown status of* S. oleracea L. *in Figure 3(c) which was treated with fermentation broth of* B. megaterium *3 times was the best in all the plates.

Response surface methodology is one more effective method that reduced development costs, optimized experimental conditions, improved production efficiency, and solved practical production problems. Compared with one-factor-at-a-time experiments, statistically designed experiments are able to describe the effect of the interactions among the factors in linear and quadratic terms [21]. In the present study, the optimization of fermentation conditions for* B. megaterium* was divided into two phases: a screening of main effects of the selected variables and a response optimization. Reduction in the initial number of variables was carried out through Plackett-Burman design that was used to screen out three factors from six factors (industrial wastewater solubility, initial pH, inoculum dose, loaded liquid, culture temperature, and shaking speed) affecting the total number of viable bacteria as major factor, which were the loaded liquid, initial pH, and culture temperature which were taken for Box-Behnken design of response surface methodology to assess their effects on the biomass of* B. megaterium*. We used response surface methodology to further optimize the biomass of* B. megaterium* by Box-Behnken design. Response surface methodology not only helped locate the optimum levels of the most significant factors but also proved to be useful and satisfactory in this process-optimizing practice. Through these optimization experiments, the maximum biomass of* B. megaterium* at 9.05 ± 0.03 × 10^8^ Obj/mL was obtained under the optimum conditions with speed 200 rmp, precooking liquid solubility 1%, temperature 25°C, initial pH 7.4, and inoculum 8%, liquid volume 40 mL/250mL, after 24 hours.

In conclusion, the effect of loaded liquid, pH, and incubation temperature and interactions between these factors all had a significant effect on the total number of viable* B. megaterium*. After optimization of the response surface, the optimum conditions for the fermentation of* B. megaterium* using* A. bisporus* wastewater were determined to be a liquid volume of 40 mL/250 mL, pH of 7.4, temperature of 25°C, precooking liquid solubility of 1%, inoculum volume of 8%, shaking speed of 200 rpm, and culture time of 24 h. Under these conditions, the total number of living bacteria can reach 9.05 ± 0.02 × 10^8^ Obj/mL, satisfying the national standard (GB 20287-2006). Furthermore, the fermentation broth of* B. megaterium *could significantly improve the yield of* Spinacia oleracea L.*

## Figures and Tables

**Table 1:** The factors and levels of Plackett-Burman experiment.

Symbol	Factors	Levels	
		-1	1
A	Solubility (%)	1.00	1.25
B	pH	6.5	8.0
C	Inoculation does (%)	8	10
D	Temperature (°C)	28	36
E	Speed (rmp)	200	250
F	Loaded liquid (mL/250mL)	30	60

**Table 2:** Experimental design of steepest ascent and corresponding results.

Run	Loaded liquid (mL/250mL)	pH	Culture temperature (°C)	Total number of living *B. megaterium *(10^7^Obj/mL)
1	150	5.5	40	0.17 ± 0.01
2	120	6.0	36	0.19 ± 0.01
3	90	6.5	32	2.96 ± 0.02
4	60	7.0	28	4.79 ± 0.02
5	30	7.5	24	14.91 ± 0.03
6	15	8.0	20	0.11 ± 0.01

Data are expressed as the mean ± standard deviation (SD) of 3-batch independent experiments.

**Table 3:** Experimental design and results of Box-Behnken design.

Run	*X*_1_: Loaded liquid (mL/250mL)		*X*_2_: pH		*X*_3_: Culture temperature (°C)		Total number of living *B. megaterium *(10^7^Obj/mL)
	Code level	Real level	Code level	Real level	Code level	Real level	
1	0	30	0	7.5	0	28	7.91 ± 0.02
2	0	30	0	7.5	0	28	8.67 ± 0.03
3	0	30	-1	6.5	-1	24	1.02 ± 0.02
4	0	30	0	7.5	0	28	8.50 ± 0.02
5	1	60	0	7.5	1	32	3.41 ± 0.02
6	1	60	1	8.0	0	28	1.12 ± 0.01
7	-1	15	-1	6.5	0	28	4.03 ± 0.02
8	-1	15	0	7.5	1	32	5.41 ± 0.03
9	-1	15	1	8.0	0	28	6.04 ± 0.03
10	1	60	0	7.5	-1	24	0.25 ± 0.02
11	0	30	0	7.5	0	28	8.57 ± 0.03
12	0	30	0	7.5	0	28	8.75 ± 0.04
13	0	30	1	8.0	1	32	4.62 ± 0.02
14	0	30	1	8.0	-1	24	1.63 ± 0.01
15	1	60	-1	6.5	0	28	2.04 ± 0.02
16	-1	15	0	7.5	-1	24	2.62 ± 0.01
17	0	30	-1	6.5	1	32	4.40 ± 0.02

Data are expressed as the mean ± standard deviation (SD) of 3-batch independent experiments.

**Table 4:** Experimental design and results of Plackett-Burman.

Run	A	B	C	D	E	F	Total number of living *B. megaterium *(10^7^Obj/mL)
1	-1	-1	1	-1	1	1	3.66 ± 0.02
2	1	1	-1	-1	-1	1	8.12 ± 0.04
3	1	1	-1	1	1	1	8.49 ± 0.03
4	1	1	1	-1	-1	-1	8.37 ± 0.04
5	-1	1	1	-1	1	1	7.52 ± 0.02
6	-1	1	1	1	-1	-1	7.16 ± 0.03
7	-1	-1	-1	1	-1	1	6.28 ± 0.02
8	1	-1	1	1	1	-1	7.38 ± 0.01
9	-1	-1	-1	-1	-1	-1	8.63 ± 0.04
10	1	-1	-1	-1	1	-1	3.08 ± 0.01
11	1	-1	1	1	-1	1	6.08 ± 0.02
12	-1	1	-1	1	1	-1	5.78 ± 0.03

Data are expressed as the mean ± standard deviation (SD) of 3-batch independent experiments.

**Table 5:** Analysis of variance in Plackett-Burman.

Source	Sum of Squares	df	Mean Squares	F Value	*p*-value	Coefficient Estimate	Importance
					Prob>F		
Model	33.00	6	5.50	7.17	0.0236		
A	0.52	1	0.52	0.67	0.4491	0.21	5
B	8.89	1	8.89	11.60	0.0191	0.86	2
C	0.27	1	0.27	0.35	0.5808	0.15	6
D	6.35	1	6.35	8.28	0.0347	-0.73	3
E	3.34	1	3.34	4.35	0.0913	-0.53	4
F	13.63	1	13.63	17.78	0.0084	-1.07	1
Residual	3.83	5	0.77				
Cor Total	36.83	11					

**Table 6:** Analysis regression and variance results in Box-Behnken design.

Source	Sum of Squares	df	Mean Squares	F Value	*p*-value	Significance
					Prob>F	
Model	142.05	9	15.78	86.36	<0.0001	*∗∗*
*X*_1_	15.90	1	15.90	87.02	<0.0001	*∗∗*
*X*_2_	0.46	1	0.46	2.52	0.1563	*∗∗*
*X*_3_	18.97	1	18.97	103.81	<0.0001	*∗*
*X*_1_ *X*_2_	2.15	1	2.15	11.74	0.0110	*∗*
*X*_1_ *X*_3_	0.034	1	0.034	0.19	0.6782	
*X*_2_ *X*_3_	0.038	1	0.038	0.21	0.6621	
*X*_1_^2^	28.11	1	28.11	153.80	<0.0001	*∗∗*
*X*_2_^2^	28.22	1	28.22	154.39	<0.0001	*∗∗*
*X*_3_^2^	37.23	1	37.23	203.73	<0.0001	*∗∗*
Residual	1.28	7	0.18			
Lack of Fit	0.84	3	0.28	2.52	0.1964	
Pure Error	0.44	4	0.11			
Cor Total	143.33	16				
R^2^=0.9911, R^2^_Adj_=0.9796, adequate precision = 26.229, CV = 9.20%						

Notes: *∗* means significant; *∗∗* means very significant.

**Table 7:** The effect of fermentation broth and *A. bisporus* industrial wastewater on the yield, plant height, and leaf area of spinach.

Group	Item		
	Yield(g/plate)	Plant height(cm)	Leaf area(cm^2^/plant)
A	228.8 ± 1.9	21.3 ± 1.2	50.1 ± 0.9
B	233.4 ± 2.5	24.4 ± 0.7	55.1 ± 1.3
C	250.1 ± 1.5*∗*	26.9 ± 0.9*∗*	59.3 ± 1.3*∗*
D	279.8 ± 4.8*∗∗*	27.9 ± 0.8*∗∗*	62.5 ± 0.8*∗∗*
E	229.7 ± 2.7	23.2 ± 1.8	53.6 ± 2.1
F	242.2 ± 2.9*∗*	25.4 ± 1.4*∗*	56.9 ± 2.3*∗*
G	259.6 ± 3.1*∗∗*	26.3 ± 1.2*∗∗*	60.1 ± 2.5*∗∗*

Notes: *∗* means significant; *∗∗* means very significant. A: control group treated with water; B: spinach treated with fermentation broth one time; C: spinach treated with fermentation broth two times; D: spinach treated with fermentation broth three times; E: spinach treated with *A. bisporus* industrial wastewater one time; F: spinach treated with *A. bisporus* industrial wastewater two times; G: spinach treated with *A. bisporus* industrial wastewater three times.

## Data Availability

The data used to support the findings of this study are included within the article. The detailed original data are available upon request from the corresponding author.
